# Global Genetic Differentiation in a Cosmopolitan Pest of Stored Beans: Effects of Geography, Host-Plant Usage and Anthropogenic Factors

**DOI:** 10.1371/journal.pone.0106268

**Published:** 2014-09-02

**Authors:** Midori Tuda, Kumiko Kagoshima, Yukihiko Toquenaga, Göran Arnqvist

**Affiliations:** 1 Laboratory of Insect Natural Enemies, Division of Agricultural Bioresource Sciences, Department of Bioresource Sciences, Faculty of Agriculture, Kyushu University, Fukuoka, Japan; 2 Institute of Biological Control, Faculty of Agriculture, Kyushu University, Fukuoka, Japan; 3 Faculty of Agriculture, Kyushu University, Fukuoka, Japan; 4 Faculty of Life and Environmental Sciences, University of Tsukuba, Tsukuba, Ibaraki, Japan; 5 Animal Ecology, Department of Ecology and Evolution, Evolutionary Biology Centre, University of Uppsala, Uppsala, Sweden; University of Lausanne, Switzerland

## Abstract

Genetic differentiation can be promoted allopatrically by geographic isolation of populations due to limited dispersal ability and diversification over time or sympatrically through, for example, host-race formation. In crop pests, the trading of crops across the world can lead to intermixing of genetically distinct pest populations. However, our understanding of the importance of allopatric and sympatric genetic differentiation in the face of anthropogenic genetic intermixing is limited. Here, we examined global sequence variation in two mitochondrial and one nuclear genes in the seed beetle *Callosobruchus maculatus* that uses different legumes as hosts. We analyzed 180 samples from 42 populations of this stored bean pest from tropical and subtropical continents and archipelagos: Africa, the Middle East, South and Southeast Asia, Oceania and South America. For the mitochondrial genes, there was weak but significant genetic differentiation across continents/archipelagos. Further, we found pronounced differentiation among subregions within continents/archipelagos both globally and within Africa but not within Asia. We suggest that multiple introductions into Asia and subsequent intermixing within Asia have generated this pattern. The isolation by distance hypothesis was supported globally (with or without continents controlled) but not when host species was restricted to cowpeas *Vigna unguiculata*, the ancestral host of *C. maculatus*. We also document significant among-host differentiation both globally and within Asia, but not within Africa. We failed to reject a scenario of a constant population size in the recent past combined with selective neutrality for the mitochondrial genes. We conclude that mitochondrial DNA differentiation is primarily due to geographic isolation within Africa and to multiple invasions by different alleles, followed by host shifts, within Asia. The weak inter-continental differentiation is most likely due to frequent inter-continental gene flow mediated by human crop trade.

## Introduction

Genetic differentiation can be induced allopatrically by geographic isolation of populations and/or sympatrically by, for example, host-race formation. In crop pests, the trade of crops across the world tends to generate a global mixing of genetic variants in pest populations, whether these were generated and maintained either allopatrically (e.g., [1],[2]) or sympatrically (e.g., [3],[4]). However, the relative contributions of allopatric or sympatric genetic differentiation versus global genotypic intermixing by natural [5] or human-aided [6]–[8] dispersal to global (continental and island) population structures has rarely been evaluated quantitatively (but see [9],[10]).

Association with specific host plants may allow the formation of genetically distinct pest host races in some cases [6], but not in others [5]. It has been suggested that an increased use of dry crops may have led to the multivoltine life cycles, polyphagy and expanded geographic distribution of stored bean pests [11]–[16]. Among the stored bean pests are several seed beetle species of the genus *Callosobruchus* (Coleoptera: Chrysomelidae: Bruchinae). Bruchine adults of such species lay eggs on dry seeds of legume crops and larvae burrow into them. Low levels of infestation are difficult to detect because these beetles live inside the seeds as larvae, pupae and pre-emergence adults. Because of the cryptic feeding in grains, world trade of dry beans for human consumption can potentially lead to the effective spread of these beetles.

In general, flight distance is correlated with body size in animals [17]. Among insects, beetles are generally weak flyers. For small beetles like seed beetles, the flight distance is limited [18] and long-distance transoceanic dispersal, even via air currents, is considered virtually impossible [19]–[21]. The low water content of stored beans is known to increase the frequency of the flightless (sedentary) form in *Callosobruchus* populations [22],[23], thus further limiting the dispersal of populations of these beetles.

*Callosobruchus maculatus* (F.) is a major stored bean pest in sub-Saharan Africa [24],[25]. It is a model organism for studies of behavior, polymorphism, population ecology, and evolutionary biology (e.g., [26]–[44]). It is considered to be of Afrotropical origin [45]. Its main, and probably ancestral, host is the cowpea *Vigna unguiculata* (L.) Walp., but other species of *Vigna* and other cultivated legumes are also infested by the beetle [16],[45]. Recent molecular work has revealed that *C. maculatus* populations are partially intermixed across agro-ecological zones of Western Africa [46].

The cowpea is a drought-tolerant food crop for humans and livestock feed that is grown throughout the semi-arid tropics [47]–[49]. It originated in southeast Africa and expanded its distribution westward and southward [50],[51]. Its domestication is estimated to have taken place in Western Africa based on maximum diversity of cultivated cowpeas [50]–[52]. It has been estimated that it was brought into India about 2,200 years ago and it has been cultivated widely in China, India and Southeast Asia [49] since. In the 17th century it was introduced to the tropical America by the Spaniards and is now widely cultivated in the United States, Caribbean islands and Brazil [49]. *Callosobruchus maculatus* also uses other economically important bean species as hosts, such as *V. radiata* and *V. angularis* [35],[37],[53], *Vicia faba*, *Cicer arietinum* and *Cajanus cajan* [16],[45],[54],[55]. Although less important as a crop, the bambara groundnut *V. subterranea* of Afrotropical origin [51] is also attacked by the beetle.

Here, we aimed to study the effects of geographic isolation by limited dispersal ability and genetic differentiation over time, host-plant utilization by formation of distinct host-associated races, and anthropogenic gene flow by trade of crop on the global genetic differentiation of *C. maculatus*, based on multiple mitochondrial and nuclear gene sequences of 42 populations that use different bean species as hosts. We evaluated these factors by comparing genetic variation between hierarchical spatial scales (continents, subregions and local populations) and between populations using different host plants. Human-aided dispersal is expected to lead to weak or no differentiation at the spatial scale where anthropogenically mediated gene flow is most frequent. We tested for congruence between the *C. maculatus* phylogeny and geography on one hand and between the phylogeny and host plant usage on the other. Furthermore, nucleotide sequence variation was compared among populations across five different continents and among those using different host plant species. The isolation-by-distance hypothesis was tested globally and within continents and with host plant controlled.

## Materials and Methods

### Ethics statement

No specific permits were required for the described field studies. No specific permission was required for sampling at any locations and activity. The locations are not privately owned or protected in any way. No activity during the field study involved endangered species or protected species.

### Study animals

*Callosobruchus maculatus* were collected as adults that emerged from randomly selected cultivated beans collected from local markets in various parts of Africa, the Middle East, South and Southeast Asia, Oceania and South America (Table 1). Populations collected in the mid-1990s or earlier were reared in the laboratory for various period of time (see Table 1). The beetles were preserved in acetone until used for DNA extraction. We used 180 sequences for phylogenetic inference and congruence and 160 sequences of recent sampling for other analyses (see the following sections).

**Table 1:** Collection data for the *Callosobruchus maculatus* populations.

Region	Subregion	Code	*n* c	Host species (*n* c)
	Population			
Africa	Western Africa			
	Mali^b^	Mal	4	NA
	Tamale, Ghana^b^	Gh	2	NA^a^
	Benin^b^	Ben	2	NA
	Lossa, Burkina Faso^b^	LosBF	5	NA
	Oyo, Nigeria^b^	OyNg	5	NA
	Borno, Nigeria	BoNg	3	*Vigna unguiculata*
	northern Nigeria	nNg	7	*Vigna unguiculata*
	Lomé, Togo	LomTg	6	*Vigna unguiculata*
	Nlongkak, Cameroon	Cam	3	*Vigna unguiculata*
	Democratic Republic of the Congo (Zaire)^b^	Zai	2	NA
	Central Africa			
	Uganda	Ug	3	NA
	Madagascar			
	Manjakandriana, Madagascar	MaMd	9	*Vigna unguiculata*
	Antananarivo, Madagascar	AnMd	9	*Vigna subterranea*
	Moramanga, Madagascar	MoMd	3	*Vigna subterranea*
Middle East	Middle East			
	Lattakia, Syria	Sy	2	*Pisum sativum* (1), *Cicer arietinum* (1)
	Oman	Om	3	NA
Asia	India/Sri Lanka/Nepal			
	Burdwan, India	BuIn	8	*Cajanus cajan*
	Columbo, Sri Lanka	SL	5	*Vigna unguiculata* (4), NA (1)
	Kathmandu, Nepal	Np	9	*Vigna unguiculata*
	Thailand/Myanmar			
	Yangon, Myanmar	My	2	*Vigna unguiculata*
	Bangkok, Thailand	BanTh	7	*Vigna unguiculata*
	Chom Thong, Thailand	CTTh	2	*Vigna angularis*
	Chiang Mai, Thailand	CMTh	6	*Vigna radiata* (3), *Vigna angularis* (3)
	China/Vietnam			
	Kunming, Yunnan, China	KmCh	8	*Vigna unguiculata*
	Hainan, China	HaiCh	8	*Vigna radiata*
	Thanh Hoa, Vietnam	THVi	5	*Vigna unguiculata* (4), *Vigna radiata* (1)
	Tam Ky, Vietnam	TKVi	2	*Vigna unguiculata*
	Taiwan/Japan			
	Yungkang, Tainan, Taiwan	TnTw	7	*Vigna unguiculata* (4), *Vigna radiata* (3)
	Taipei, Taiwan	TpTw	3	*Vigna radiata* (1), *Vigna angularis* (2)
	Okinawa, Japan	OkiJ	1	*Vigna unguiculata*
	Indonesia/Philippines			
	Los Banos, Laguna, Philippines	Ph	8	*Vigna radiata*
	Bali, Indonesia	Bal	8	*Vigna radiata*
	Java, Indonesia	Jav	2	*Vigna radiata*
	Kota Kinabalu, Malaysia	KK	9	*Vigna radiata*
Oceania	New Zealand			
	New Zealand	NZ	3	*Vicia faba*
South America	Brazil			
	Uberlandia, Brazil	Br	9	*Vigna unguiculata*

Locations are categorized by regions (continents or archipelagos) and by subregions.

a [101].

b excluded from dataset for AMOVA, IBD test and sudden demographic growth test because of >10 years of laboratory rearing and/or small sample sizes.

c number of individuals used.

### DNA sequencing

Whole genomic DNA was extracted from individual beetles using a DNeasy Blood & Tissue Kit (QIAgen, Tokyo, Japan). We amplified and sequenced two mitochondrial fragments, *COI* and *tRNA^Leu^–COII*, and nuclear *EF-1α* (elongation factor-1α) and two fragments from *28S rRNA*. For the PCR and sequencing, we followed the protocol previously described in [6],[14]. The primers used were COI-F (5′-ATAATTTTTTTTATAGTTATACC-3′) and COI2-2 [6] for *COI*, and 5′-TAATATGGCAGATTAGTGCATTGGA-3′ and 5′-GAGACCATTACTTGCTTTCAGTCATCT-3′ ([56], modified from [57]; see [14] for details) for *tRNA^Leu^–COII*. Nuclear *EF-1α* fragment was amplified using primers 5′-GGTATCACCATTGATATTGCHTTDTGGAA-3′ and 5′-ACCAGCAACATAACCACGACG-3′ [58]. Two nuclear *28S rRNA* gene fragments were amplified using the primers, 28S-01 (5′-GACTACCCCCTGAATTTAAGCAT-3′) in combination with 28S-R01 (5′-GACTCCTTGGTCCGTGTTTCAAG-3′) for D2–D3 fragment [59] and 28SD4-5-F in combination with 28SD4-5-R for D4–D5 fragment [60]. The PCRs were performed following [6] with a total volume of 15 µl; initial preheating at 95°C for 2 min, followed by 35–40 cycles of a denaturation phase at 94°C for 30 s, an annealing at 45°C for *COI*, 51°C for *tRNA^Leu^*–*COII*, a constant temperature between 44–52°C for *EF-1α*, 54°C for 28S-01 and 51°C for 28SD for 40 s, and an extension at 60°C for the mitochondrial genes, 62°C for the *EF-1α* and at 70°C for the *28S* fragments, for 1 min–1 min 10 s. Sequencing was done with BigDye Terminator v3.1 Cycle Sequencing Kit (Life Technologies/Applied Biosystems) on 3730 DNA Analyzer (Applied Biosystems).

Because infection by and vertical transmission of the intracellular symbiont *Wolbachia* may select for mitochondrial haplotypes in infected females through cytoplasmic incompatibility [61], we used PCR to diagnose possible *Wolbachia* infection using primers fts-Z-f and fts-Z-r for the *ftsZ* gene coding fragment [62]. Here, beetles of *Callosobruchus chinensis* (L.) that were naturally infected with *Wolbachia* were used as a positive control [63].

### Reconstruction of molecular phylogeny

The phylogenetic relationships among the beetle populations were estimated by Bayesian inference using MrBayes 3.2 [64],[65]. *Callosobruchus imitator* Kingsolver (Genbank accessions DQ459044, DQ459028) and *C. subinnotatus* (Pic) (DQ459047, DQ459031) were used as outgroups (see [14]). An evolutionary model for reconstruction of the molecular phylogeny for the populations was selected from the 24 models in MrBayes 3.2 [65] using MrAIC.pl 1.3.1 [66]. For independent gene trees, the HKY+Γ model (Hasegawa, Kishino and Yano model [67], with a gamma distribution of rates across sites) was supported both for *COI* and for *tRNA^Leu^*–*COII* by AICc. For all model parameters, we used the default priors. The mitochondrial gene sequence data were subjected to two independent parallel runs each with four Metropolis-coupled chains [68] of which three were heated (i.e., accepted at a higher rate than specified by the Metropolis-Hastings criterion) using MCMC (Markov chain Monte Carlo, [69],[70]) algorithms, sampled every 1,000 generations. The convergence of parameters among runs was checked using Tracer 1.5.0 [71]. The separate gene trees were estimated using similar parameters with 6 million generation runs. The combined data set was subjected to 10 million generation runs with a partition between *COI* and *tRNA^Leu^*–*COII*. The first 25% of the total number of generations were discarded as a burn-in phase. For comparison of congruence of phylogenetic topology with geography and host plants (see the next section), we used a subset of the data with host plant information (Table 1) and re-estimated the *C. maculatus* phylogeny using MrBayes.

### Congruence between *C. maculatus* phylogeny and geography/hosts

The congruence of the *C. maculatus* phylogeny with either the geographic subregions (West and Central Africa, Madagascar, the Middle East, India/Sri Lanka/Nepal, Thailand/Myanmar, China/Vietnam, Taiwan/Japan, Indonesia/Philippines, New Zealand, and Brazil) or host plants (*V. unguiculata*, *V. radiata*, *V. angularis*, *V. subterranea*, Vicieae (*Vicia* and *Pisum*), *Cajanus*, and *Cicer*) was tested with a topology-dependent permutation tail probability test (T-PTP) implemented in PAUP*4.0 Beta [72] for ingroup taxa only [73]. Randomization of the traits was performed 1,000 times. For this test, we used a Bayesian phylogeny that was estimated from the sequence data after excluding individuals with unknown hosts (see the previous section). We confirmed that this exclusion did not change the basic topology of the phylogeny. Nevertheless, to assure this exclusion would not affect congruence between phylogenetic topology and geography, we also tested the congruence between geography and phylogeny before the exclusion.

### Population structure

Here, we used populations of which individuals were analyzed either soon after collection or within a relatively brief rearing history in the lab (160 individuals in total; see Table 1) to minimize any effects of decrease in genetic variability during rearing. Although rearing even for several generations may erode ancestral genetic signature, the laboratory populations we used had been kept at large population sizes to avoid any bottleneck events. We performed hierarchical analyses of molecular variance (AMOVAs) [74]–[76] to compare genetic variances at different hierarchies of either geographic or host plant groups (*V. unguiculata*, *V. angularis*, *V. radiata*, *V. subterranea* and *C. cajan*; the other plant species or groups were excluded because of the small sample sizes, Table 1), using Arlequin 3.5.1.3 [77]. Furthermore, to exclude confounding effects resulting from the correlation between geographic regions and host plants, we performed AMOVAs either with geographic regions controlled or with host plants controlled. To control possible effect of unequal sample sizes among populations, we used a resampling procedure where two individuals per population were randomly sampled and subjected to AMOVAs to test statistical robustness of geographic and host-plant effects detected in the whole dataset. We used Tamura and Nei's distance [78]. To correct skewed distribution of nucleotide substitutions among sites, gamma distribution was assumed with *α* = 0.05 for the mitochondrial genes and *α* = 0.3 for the nuclear gene, based on our estimation for the *C. maculatus* dataset.

### Isolation by distance

As in the previous analyses, we only used populations that were analyzed soon after collection or within a brief rearing history. We assessed spatial genetic differentiation by testing for isolation by distance (IBD) [79]. With frequent anthropogenic long-distance dispersal events, the IBD correlation is predicted to be weak at most. We tested if, as predicted by IBD, geographic distance (ln-transformed) and *F*_ST_/(1- *F*_ST_) were positively correlated, by a one-tailed Mantel test based on 2,000 permutations, using the ISOLDE program of Genepop 4.0.10 [80],[81]. We first tested IBD on the global dataset. We then tested for IBD within Africa and within Asia to assess genetic intermixing at a smaller scale. We also restricted this analysis to include only populations using *V. unguiculata* as a host to exclude possible effect of host-race formation over distance.

### Demographic growth and selective neutrality

As in the previous two sections, we used only populations that were subjected to DNA extraction shortly after collection. Recent past sudden demographic expansion was tested in populations on different continents (Africa and Asia) and in populations using the original host *V. unguiculata*, with raggedness *r* [82], *F_S_* [83] and *R*_2_ [84], using DnaSP5.10.0.1 [85]. Nonsignificant *r*, significantly negative *F_S_* and significantly small *R*_2_ values indicate recent sudden population growth. The latter two indices are statistically more powerful than the raggedness index and *F_S_* exhibits high statistical power for large sample sizes, while *R*_2_ is more powerful for small sample sizes [84]. Selective neutrality was tested with Tajima's *D* [86], using DnaSP. Coalescent simulations were performed for 2,000 iterations to test statistical significance of these indices, using DnaSP.

## Results

### Molecular phylogeny

The total lengths of the sequenced fragments were 1,726 bp (1,005 bp for *COI*, 19 bp for *tRNA^Leu^*, and 702 bp for *COII*) for the mitochondrial genes (genbank JX297380–JX297419). The sequence data included no indels and the base composition was AT rich, as is typical for insect mitochondrial sequences. The nuclear *EF-1α* fragment was sequenced for 81 homozygous individuals. We used 866 bp of this fragment, including insertions and deletions. For the nuclear *28S*, the sequenced fragments were 1,452 bp in total (822 bp for the D2–D3 fragment and 630 bp for the D4–D5). There was no sequence variation across *C. maculatus* individuals in the two fragments of *28S* and these were thus discarded from subsequent analyses. We analyzed only the mitochondrial trees because homozygous nuclear *EF-1α* data was available for limited number of individuals.

**Figure 1:**
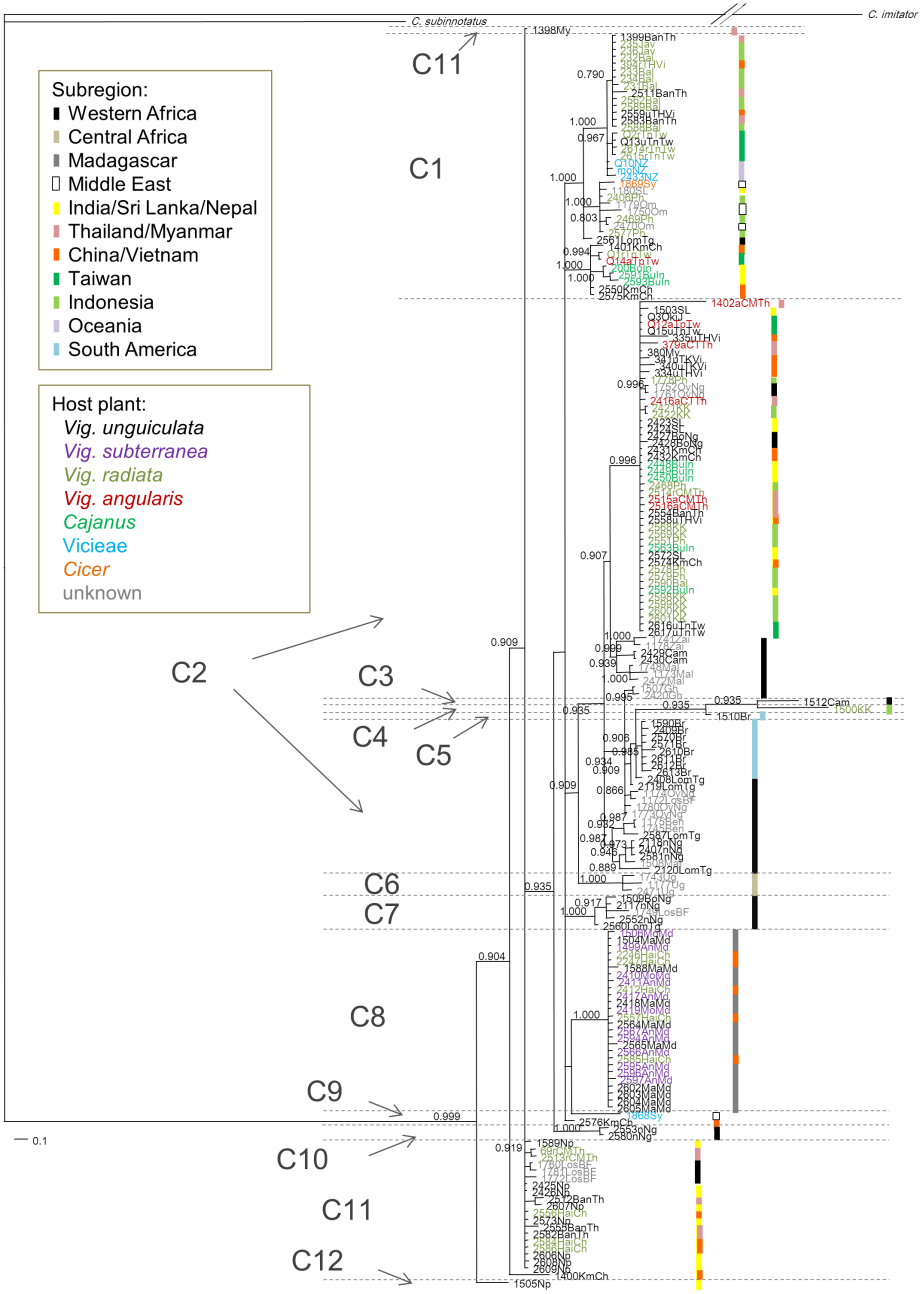
Bayesian majority rule consensus tree of *Callosobruchus maculatus* populations, based on mitochondrial genes. The numbers near the nodes indicate posterior probabilities (only probabilities >0.700 are shown). The colors of the sample codes indicate the host plants used by the populations when collected. The colors of the vertical bars indicate the geographic regions where the population samples were collected.

**Figure 2:**
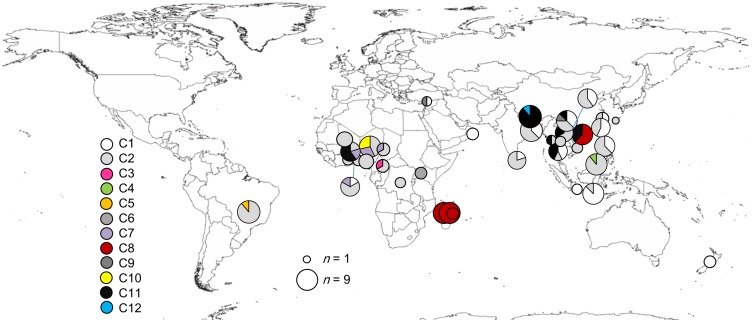
World map showing haplotypic compositions (based on clades identified in Fig. 1) of local populations. The size of pie charts indicates the number of individuals sampled.

The Bayesian majority rule consensus tree based on mitochondrial genes was polytomized (Fig. 1). Twelve clades (C1–C12), which were diverged with at least 0.5 nucleotide substitutions per site from the closest clade, were recognized. Based on these clades, there have been at least seven introductions of *C. maculatus* into Asia (clades C1, C2, C4, C8, C9, C11 and C12, Figs. 1 and 2). Separate gene trees for *COI* and *tRNA^Leu^*–*COII* and the combined tree differed in topology but the combined and the *COI* trees revealed the very same set of clades (Figs. 1 and 1).

None of the individuals showed a positive diagnostic *Wolbachia* PCR, in line with the previous finding that *Wolbachia* infection does not occur in *C. maculatus* [87].

### Congruence between *C. maculatus* phylogeny and geography/hosts

Randomization of the geographic locations changed the length of the *C. maculatus* mitochondrial phylogenetic tree (T-PTP, *P* = 0.001), indicating highly significant congruence between the beetle phylogeny and geographic regions. Inclusion of individuals without host information did not alter the significant relationship between geographic location and the *C. maculatus* phylogeny (T-PTP, *P* = 0.001). Randomization of the host bean groups also changed the length of the *C. maculatus* phylogenetic tree (T-PTP, *P* = 0.001), indicating that the congruence between the beetle phylogeny and host plant use was as significant as between phylogeny and geography.

### Population structure

Out of the 160 individuals sampled recently (Table 1), there were 93 distinct haplotypes for *COI* (gene diversity ± SD, 0.970±0.007; nucleotide diversity ± SD, 0.0282±0.0137) and, out of 91 sequences, 34 haplotypes for *tRNA^Leu^*–*COII* (gene diversity, 0.943±0.010; nucleotide diversity, 0.0165±0.0083). In total, there were 111 different mitochondrial haplotypes (gene diversity, 0.991±0.003; nucleotide diversity, 0.0136±0.0066). For the *EF-1α*, out of 69 individuals sequenced, there were 60 different alleles (gene diversity ± SD, 0.991±0.006; nucleotide diversity ± SD, 0.0344±0.0169).

The AMOVA on the mitochondrial genes revealed significant genetic differentiation among continents/archipelagos, among subregions within continents/archipelagos, and within subregions among populations (Table 2). However, when unequal sample sizes were corrected by randomly re-sampling two individuals per population, the among-continent/archipelago variation became much less pronounced (*P* = 0.016–0.072). When continents were controlled for, subregional differentiation was significant in Africa but not in Asia (Table 2). When host plant usage was restricted to only the populations using *V. unguiculata*, variation across continents was no longer significant (Table 2).

**Table 2:** Tests of genetic differentiation among and within continents/archipelagos (regions).

	AMOVA (% variance) [d.f.]				
	Global		Continent controlled		Host plants controlled (*V. unguiculata*)
	mDNA	nDNA	Africa	Asia	
Among continents or archipelagos	18.3**^a^ [4]	−8.7^NS^ [4]			8.5 ^NS^ [2]
Among subregions	15.4** [7]	24.8** a [6]	46.3** [2]	−7.4^NS^ [4]	24.2** a [4]
Among populations	66.3** [148]	83.9* [57]	21.3** a [5]	45.5** a [13]	67.3** a [71]
Within populations	-	-	32.4** [35]	61.9** [82]	-
Among host plants	17.0** [4]	2.5* a [3]	−4.0 ^NS^ [1]	31.6** [2]	-

Analyses of molecular variance (AMOVAs). Data include populations analyzed immediately after collection or reared in the lab briefly (Table 1). Unless otherwise noted as the nuclear DNA (nDNA), the mitochondrial DNA (mtDNA) sequence data were used.

*P<0.05, **P<0.01, NS: *P*>0.05.

a The statistical significance was not robust to equalizing the sample sizes per population (see text).

The AMOVA indicated genetic differentiation among populations using different host-plant species globally for mitochondrial DNA (Table 2). However, host plant usage and geographic area were associated. Thus, we controlled for continental effects and re-analyzed the data. When geography was thus controlled for, there was no significant genetic differentiation among populations using different host species for African populations (Madagascar). However, significant variation was found among populations using different host plants for Asian populations (Chiang Mai, Taipei, Tainan, and Thanh Hoa, where populations were sampled from multiple host species) (*P* = 0.0089, Table 2). When all Asian populations were included, the variation across populations remained significant among populations using different host plants (variation 9.8% of total variation, d.f. = 3, *P* = 0.0029). None of these results changed, in terms of our ability to reject null hypotheses, when unequal sample sizes per population were controlled for.

In contrast to the results based on mitochondrial DNA, the AMOVA of the nuclear gene showed no genetic differentiation among continents. Genetic differentiation among populations using different host plants was significant as in mitochondrial genes but this effect was no longer present when the sample size was controlled for (Table 2).

### Isolation by distance

Geographic distance (ln-transformed) and *F*_ST_/(1- *F*_ST_) between pairwise populations were significantly correlated (Mantel test *P* = 0.0020, Table 3), supporting the hypothesis that IBD forms at least one component of population differentiation. The correlation was significant both within Africa (*P* = 0.0305, Table 3) and within Asia (*P* = 0.0410, Table 3). When host plants were restricted to *V. unguiculata*, however, the global correlation was marginally non-significant (*P* = 0.0545, Table 3).

**Table 3:** Tests of the isolation by distance hypothesis (IBD) for the mitochondrial genes.

	IBD (*r_S_*) [*n*]			
	Global (all regions and host plants included)	Continent controlled		Host plants controlled (*V. unguiculata*)
		Africa	Asia	
Population	0.308** [434]	0.437* [28]	0.252* [152]	0.243 ^NS^ [105]

Mantel test *P*, *:<0.05, **:<0.01, NS: *P*>0.05. *n*: number of pairwise comparisons.

### Demographic growth and selective neutrality

Since our sample sizes were large, we focused on the *F_S_*. We failed to reject a scenario of population stability in the recent past combined with selective neutrality for the mitochondrial genes for global, African or Asian populations and for the populations using either *V. unguiculata* or other hosts (Table 4). In contrast, for nDNA, selective neutrality was rejected for the global, African, or Asian population and for the population using *V. unguiculata* (Table 5). The model of a recent past constant population size was rejected for global and African populations for nDNA, indicating demographic growth on the global scale and in Africa in *C. maculatus* (Table 5). When host plant was controlled to either *V. unguiculata* or other hosts, recent past global population growth in *C. maculatus* was no longer supported (Table 5). The number of segregating (polymorphic) sites was higher for African populations than for Asian populations and for populations using *V. unguiculata* than those using hosts other than *V. unguiculata*, for nDNA (Table 5).

**Table 4:** Tests of sudden demographic growth in the recent past for the mitochondrial genes.

	Global	Continent		Host plant	
		Africa	Asia	*V. unguiculata*	Other hosts
*n*	158	43	99	78	73
*S*	81	50	88	60	88
*D*	−0.511 ^NS^	−0.708 ^NS^	−0.518 ^NS^	−0.236 ^NS^	−0.641 ^NS^
Raggedness *r*	0.0892 ^NS^	0.1026 ^NS^	0.0587^NS^	0.0642 ^NS^	0.0892 ^NS^
*F_S_*	−0.43 ^NS^	0.59 ^NS^	4.82 ^NS^	0.49 ^NS^	6.57 ^NS^
*R*_2_	0.0767 ^NS^	0.0913^NS^	0.0855^NS^	0.0985 ^NS^	0.0858 ^NS^

NS: *P*>0.05.

*n*: number of individuals.

*S*: number of segregating (polymorphic) sites.

*D*: Tajima's *D* [86].

Raggedness *r*: [82].

*F_S_*: [83].

*R*_2_: [84].

**Table 5:** Tests of sudden demographic growth in the recent past for the nuclear gene.

	Global	Continent		Host plant	
		Africa	Asia	*V. unguiculata*	Other hosts
*n*	68	23	36	28	37
*S*	11	50	33	82	9
*D*	−1.630*	−1.974**	−2.015**	−2.058**	−1.145 ^NS^
Raggedness *r*	0.0688 ^NS^	0.0130*	0.0198*	0.0158 ^NS^	0.0519 ^NS^
*F_S_*	−6.38**	−5.99*	−0.53 ^NS^	−3.82 ^NS^	−2.39 ^NS^
*R*_2_	0.0461*	0.0556***	0.0966 ^NS^	0.0570**	0.0744 ^NS^

NS: *P*>0.05, *:<0.05, **:<0.01, ***:<0.001.

*n*: number of individuals

*S*: number of segregating (polymorphic) sites.

*D*: Tajima's *D* [86].

Raggedness *r*: [82].

*F_S_*: [83].

*R*_2_: [84].

## Discussion

We aimed to study the relative contribution of factors associated with geography, host plant usage and human-aided dispersal to genetic population structure of the stored bean pest, *C. maculatus*, mainly in Africa and Asia. On a continental scale, there was among-subregional genetic variation within Africa but not within Asia. Among-host genetic population differences were found only in Asian *C. maculatus* populations. The IBD hypothesis was supported globally, but not for the populations using *V. unguiculata*, the main host of *C. maculatus*. The number of segregating sites was higher for populations in Africa than in Asia and for those using hosts other than *V. unguiculata* than those using *V. unguiculata* for the nuclear gene, supporting the hypothesis that Africa is the region of origin for *C. maculatus* [45]. We suggest that the differentiation among hosts in *C. maculatus* is due to multiple invasions on novel hosts by different alleles, particularly in Asia. Geographic effects [8] that are larger than host-plant effects [10] have also been reported in cosmopolitan moths, the apple moth and the fruit moth, although among-continental differentiation seems more pronounced in those pests than in *C. maculatus* (Table 2). This may be partly due to a higher frequency of anthropogenic cross-continental dispersal in *C. maculatus*, aided by human trade of host beans than in these moths. Different levels of dispersal may be generated by differences in the frequency of inter-continental trade, in pest control efficiency and in pest survival during transport (fresh, soft, decaying fruits in moths versus dry, hard seeds in *C. maculatus*).

### Geographic structuring

We found that there was genetic structure among African *C. maculatus* subregional populations and suggest that this most likely represents allopatric divergence, because the structure primarily reflects the geographic separation of populations (Figs. 1 and 2, Table 2). This is likely mainly the result of historical differentiation along with natural and human-aided dispersal [88] and more recent international trade of dry cowpeas, *V. unguiculata*, which has been restricted mostly to within Western Africa [49]. We note that it is also possible that some degree of local adaptation and/or reproductive incompatibility between different geographic populations of *C. maculatus* has contributed to the maintenance of geographic genetic structure. Asian populations show limited mating incompatibility (with African populations [89],[90]) as well as some prezygotic postmating incompatibility (with a Brazilian population [91]).

We could not reject the hypothesis that the mtDNA variation documented here is primarily due to genetic drift (Table 4). This may seem surprising given that phenotypic effects of mtDNA variation have been demonstrated in *C. maculatus* [40],[92] and in many other species [93],[94]. However, geographically varying natural selection regimes, caused for example by differences in thermal environments, could contribute to patterns such as that documented here. Moreover, the fact that within-population mtDNA haplotype variation was found to be fairly high and significant (Table 2) is interesting in light of recent experimental work in *C. maculatus* demonstrating that negative frequency-dependent selection acts to maintain within-population genetic variation of mtDNA haplotypes in the laboratory [95]. Although our data do not allow for formal tests of frequency dependent selection, this finding suggests that natural selection may play a role in differentiation of mitochondrial genetic variation.

### Host plant race

Host race formation likely contributes to the maintenance of genetic structure in invaded areas like Asia (Table 2). However, host acceptance of *C. maculatus* is broad and evolves rapidly in the laboratory (e.g., [34],[96],[97]) and this broad and labile host acceptance may impede the formation of rigid host-races. The high genetic diversity among populations using different hosts in Asia is thus intriguing. One might also argue that selection and cultivation of Asian yardlong bean, *V. unguiculata* var. *sesquipedalis* could have contributed to the genetic differentiation of this seed-predator in Asia. However, seedpods of yardlong beans are harvested at a young stage to use as a fresh vegetable [49], unlike other *V. unguiculata* varieties that are harvested at later mature stages for dry seeds, and this most likely limits infestation by postharvest pests such as *C. maculatus*. We suggest that the differentiation among populations using different host plants in Asia may be the combined result of multiple invasions by different alleles followed by host-shifting to Asian legumes, although it is difficult to exclude a role of selection. The reduced cultivation of *V. unguiculata* relative to other beans in Asia likely decreased the availability of *V. unguiculata* for *C. maculatus* and may increase the chance of exploration and further differentiation on alternative hosts.

### Anthropogenic dispersal

Overall, genetic differentiation in *C. maculatus* was primarily due to geographic isolation and multiple cross-continental anthropogenic dispersal events rather than to the formation of strict host-plant races. More frequent within-continental international trade of dry beans likely reduced the subregional genetic differentiation in Asia more than in Africa. In spite of the overall geographic structuring, some Asian populations shared similar haplotypes with African populations (e.g., Hainan and Madagascar populations, clade C8; India, Sri Lanka, Myanmar, Thailand, China, Japan, Taiwan, Philippines and Nigeria, clade C2 in Figs. 1 and 2). This may represent examples of more recent introductions into Asia from Africa. Three million out of 3.6 million tonnes of annual world production of dry cowpeas were produced in Western Africa during 1999–2003, the main producer being Nigeria [47],[49],[98]. Since the mid-1990s, Africa has exported/imported negligible amounts of dried cowpeas (Fig. S2, [97]). Furthermore, about 80% of the world cowpea trade takes place within Western and Central Africa [99]. Likewise, international trade of dry cowpeas and other *Vigna* beans within Asia probably masked any effect of geographic isolation (e.g., between ASEAN countries [Association of Southeast Asian Nations], the main exporter being Myanmar, [97],[100]).

To conclude, we found significant subregional-scale genetic differentiation in Africa and among-host difference in Asia. We suggest that this contrasting pattern has primarily been caused by allopatric differentiation in Africa and by multiple invasions by different alleles, followed by host shifts in Asia. Recent long-range human trade of dry beans has allowed dispersal from Africa to other continents and medium-range trade has subsequently intermixed the introduced alleles within Asia. In Africa, intermixing has occurred at a smaller spatial scale.

## Supporting Information

Figure S1 **Bayesian majority rule consensus tree of** ***Callosobruchus maculatus*** **populations, based only on the mitochondrial** ***COI*** **gene.** The numbers near the nodes indicate posterior probabilities (only probabilities >0.700 are shown).
(TIF)

Figure S2 **Export and import quantities of dry cowpeas (tonnes) in different African and Asian subregions.** Data on imports into South Asia were unavailable.
(TIF)
