# Supplementary figures and images for: Global Genetic Differentiation in a Cosmopolitan Pest of Stored Beans: Effects of Geography, Host-Plant Usage and Anthropogenic Factors
Source: PLoS One. 2014 Sep 2;9(9):e106268. doi: 10.1371/journal.pone.0106268 (PMC4152179; doi:10.1371/journal.pone.0106268)

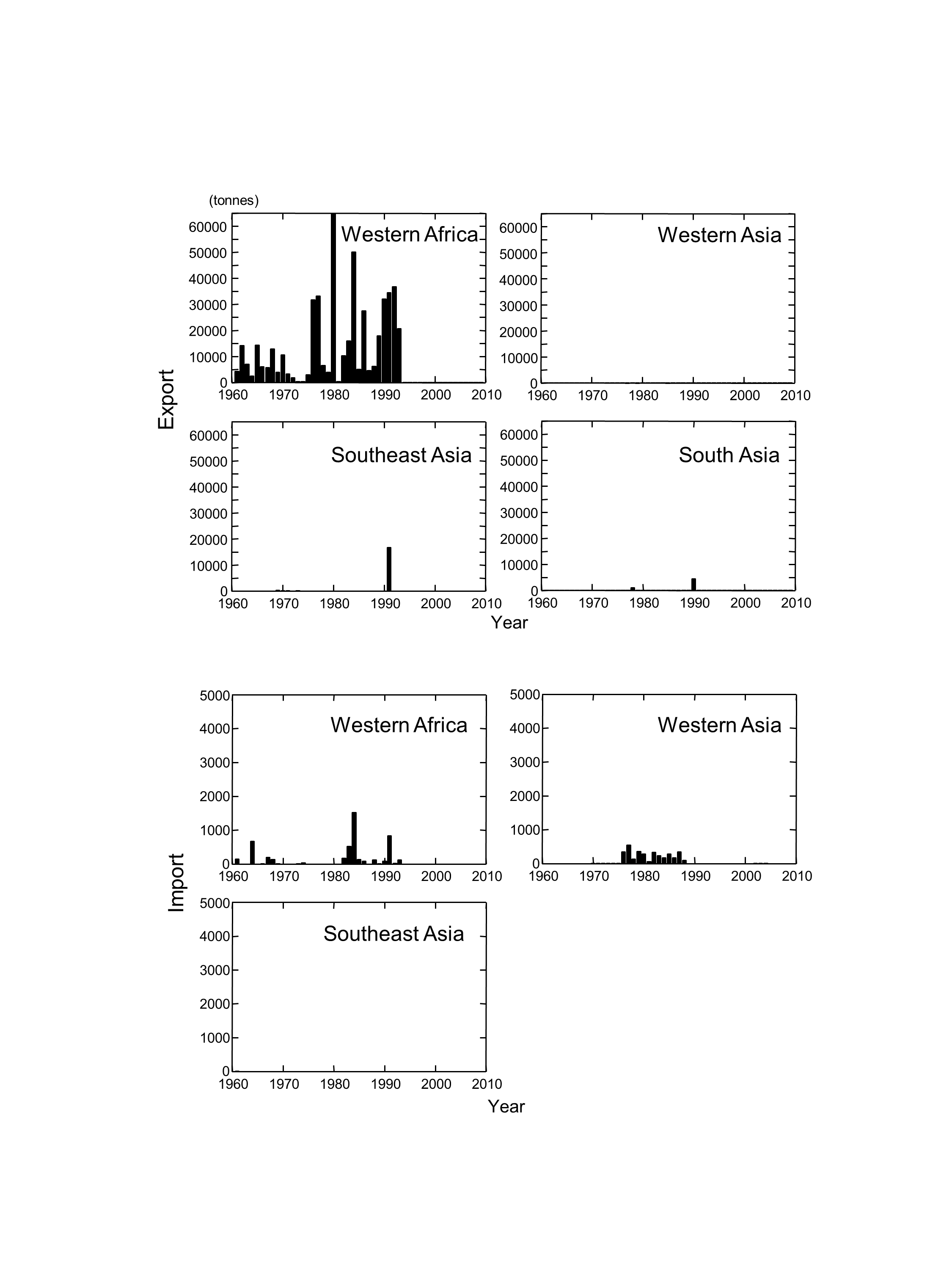

Supplement: Figure S2 — Export and import quantities of dry cowpeas (tonnes) in different African and Asian subregions. Data on imports into South Asia were unavailable. (TIF) [file pone.0106268.s002.tif]
